# Prevention Effects and Possible Molecular Mechanism of Mulberry Leaf Extract and its Formulation on Rats with Insulin-Insensitivity

**DOI:** 10.1371/journal.pone.0152728

**Published:** 2016-04-07

**Authors:** Yan Liu, Xuemei Li, Chen Xie, Xiuzhen Luo, Yonggang Bao, Bin Wu, Yuchi Hu, Zhong Zhong, Chang Liu, MinJie Li

**Affiliations:** 1 Research and Development Center of Amway (China), Shanghai, China; 2 Beijing Institute for Drug Control (Beijing Center For Health Food And Cosmetics Control), Beijing, China; 3 Botanic Century (Beijing) Co., Ltd, Beijing, China; 4 Beijing Pepnoch Co., Ltd, Beijing, China; 5 Institute of Medicinal Plant Development, Chinese Academy of Medical Sciences and Peking Union Medical College, 151 Malianwa North Road, Haidian District, Beijing 100193, China; USDA-ARS, UNITED STATES

## Abstract

For centuries, mulberry leaf has been used in traditional Chinese medicine for the treatment of diabetes. This study aims to test the prevention effects of a proprietary mulberry leaf extract (MLE) and a formula consisting of MLE, fenugreek seed extract, and cinnamon cassia extract (MLEF) on insulin resistance development in animals. MLE was refined to contain 5% 1-deoxynojirimycin by weight. MLEF was formulated by mixing MLE with cinnamon cassia extract and fenugreek seed extract at a 6:5:3 ratio (by weight). First, the acute toxicity effects of MLE on ICR mice were examined at 5 g/kg BW dose. Second, two groups of normal rats were administrated with water or 150 mg/kg BW MLE per day for 29 days to evaluate MLE’s effect on normal animals. Third, to examine the effects of MLE and MLEF on model animals, sixty SD rats were divided into five groups, namely, (1) normal, (2) model, (3) high-dose MLE (75 mg/kg BW) treatment; (4) low-dose MLE (15 mg/kg BW) treatment; and (5) MLEF (35 mg/kg BW) treatment. On the second week, rats in groups (2)-(5) were switched to high-energy diet for three weeks. Afterward, the rats were injected (ip) with a single dose of 105 mg/kg BW alloxan. After four more days, fasting blood glucose, post-prandial blood glucose, serum insulin, cholesterol, and triglyceride levels were measured. Last, liver lysates from animals were screened with 650 antibodies for changes in the expression or phosphorylation levels of signaling proteins. The results were further validated by Western blot analysis. We found that the maximum tolerance dose of MLE was greater than 5 g/kg in mice. The MLE at a 150 mg/kg BW dose showed no effect on fast blood glucose levels in normal rats. The MLE at a 75 mg/kg BW dose and MLEF at a 35 mg/kg BW dose, significantly (p < 0.05) reduced fast blood glucose levels in rats with impaired glucose and lipid metabolism. In total, 34 proteins with significant changes in expression and phosphorylation levels were identified. The changes of JNK, IRS1, and PDK1 were confirmed by western blot analysis. In conclusion, this study demonstrated the potential protective effects of MLE and MLEF against hyperglycemia induced by high-energy diet and toxic chemicals in rats for the first time. The most likely mechanism is the promotion of IRS1 phosphorylation, which leads to insulin sensitivity restoration.

## Introduction

Prediabetes and diabetes are prevalent in modern societies worldwide. The treatment for diabetes is expensive and causes significant burden for both the patients’ family and the society. Herbal intervention could be an effective and safe approach to prevent diabetes development and to improve the life quality of diabetes-susceptible population. In Asian countries, herbal ingredients are traditionally used for the prevention or treatment of prediabetes and diabetes. However, herbal extracts usually contain hundreds of chemical compounds. Identification of those anti-hyperglycemic active compounds has been difficult. Furthermore, the underlying mechanisms were not usually elucidated. As a result, the assurance of constant safety and efficacy of herbal extracts has also been difficult. This outcome largely limited their wide range of applications in diabetes prevention and treatment.

Recent studies on mulberry leaf have demonstrated its physiological activities on various metabolic diseases, such as cardiovascular diseases [1], obesity [2], diabetes [3, 4], and hyperlipidemia [5]. 1-Deoxynojirimycin (DNJ) is regarded as one of the major active iminosugars in mulberry leaf extracts (MLE). Notably, MLEs used in previous studies have several drawbacks. First, the DNJ content is low. As a result, large dose of the extracts has to be consumed to ensure efficacy. Second, those MLEs usually have a dark color and unpleasant odor. These unfavorable properties have limited the applications of MLEs. Previously, we have developed a process to obtain MLEs that have a higher DNJ content and lighter color, odor, and taste, which can be used at a lower dose and can be easily added into health care and food products. The current study is intended to test the safety and efficacy of this MLE preparation.

In addition to MLE, recent studies have reported the anti-hyperglycemic activities of fenugreek seeds and cinnamon. 4-Hydroxyisoleucine, as a unique compound in the fenugreek seeds, has been reported to stimulate glucose uptake [6], improve insulin resistance [7], and have insulinotropic effect [8, 9]. By contrast, polyphenol is considered one of the important classes of active compounds in cinnamon extract, which was reported to have a similar glucose uptake stimulation effect [10, 11]. This substance was also reported to enhance insulin receptor sensitivity to insulin stimulation [12]. In addition, polyphenol has an in vitro stimulatory effect of insulin release [13]. To maximize the potential synergistic effects of these three herbal extracts, MLEF was formulated through the combination of MLE with 4-hydroxyisoleucine-enriched fenugreek seed extracts and polyphenol-enriched cinnamon cassia extracts.

In the current study, an animal model with hyperglycemia induced by high-fat diet and alloxan injection was used to observe the anti-hyperglycemic effects of MLE and MLEF. Two animal groups were treated with MLE at a low dose (15 mg/kg BW) and a high dose (75 mg/kg BW) separately. In parallel, to observe the possible synergistic effects of MLE, another group of rats was treated with MLEF at a 35 mg/kg BW dose, which contains 15 mg/kg BW MLE. Finally, the possible underlying mechanisms were explored.

## Materials and Methods

### 2.1 Materials

Acetonitrile of HPLC grade was purchased from J. T. Baker (USA). Ammonia (A.R), ammonium acetate (A.R), and ethanol (A.R) were acquired from Beijing Chemical works (China). Cation exchange resin (001*7) and macroporous resin (AB-8) were obtained from The Chemical Plant of Nankai University (Tianjin, China).

Animal feeds were purchased from Beijing Keaoxieli Animal Feed Company. Glucose (Glu) test kit (batch no.: 120521), triglyceride (TG) test kit (batch no.: 120631), and total cholesterol (CHO) test kit (batch no.: 120961) were purchased from Zhongsheng Beikong Biotech Co., Ltd. (Beijing, China). Alloxan (batch no.: 39H1482) was purchased from Sigma. Glucose (batch no.: 040302) was purchased from Beijing chemical reagents company (China). Rat serum insulin assay kit (batch no.: 20120701A) was purchased from R&D (MN, USA).

Roche complete mini protease inhibitor cocktail tablet (catalog no: 11836153001) was purchased from Roche (Indianapolis, USA). Phenylmethylsulfonyl fluoride (catalog no: 36978) was purchased from Thermo fisher. Leupeptin (catalog no: 04906837001) is from Roche. Pierce BCA protein assay kit (catalog no. 23227, Thermo Scientific). Mouse anti-GAPDH monoclonal antibodies and goat anti-rabbit or mouse immunoglobulin G (IgG) conjugated with horseradish peroxidase were purchased from Beijing Tiandeyue Biotech Co., Ltd. (China).

### 2.2 MLE/MLEF preparation and quality control

Leaves of *Morus alba* L. were collected from Anhui Province, China. Plant identity was verified by Professor Yulin Lin from Institute of Medicinal Plant (IMPLAD), Chinese Academy of Medical Science. In addition, the voucher specimen was kept in the IMPLAD herbarium. MLE was prepared by the method described below.

The leaves were dried under 23°C to 25°C at 20% to 30% humidity for 3 days. After the two extractions of dried leaves with 10 times water by weight, the combined extraction solution was passed through a strong cation exchange chromatography column. After washing with water, the 0.5 M amino solution elution was collected and concentrated by vacuum evaporator to obtain the alkaline fraction. This fraction was re-dissolved in water and passed through a macroporous resin column. Afterward, the effluent was collected, concentrated, and spray dried to obtain MLE.

Analyses of MLE DNJ content were performed using Waters HPLC system, which consists of a 600 pump and a 2420 ELS detector. The ELSD detector was set as spray temperature at 60%, drift tube temperature at 50°C, gain value at 200, and nitrogen flow at 25 psi. The separation was performed on a Shodex ^®^ Asahipak NH2P-50 4E column (4.6 mm * 250 mm) and the column temperature was set at 30°C. Acetonitrile–water, which contains 6.5 mM ammonium acetate, of 84:16 ratio by volume was used in the mobile phase, and the flow rate was 1 mL/min.

MLEF was formulated by Amway (China) R&D Ltd. This formula was prepared through the mixture of MLE with the commercially available cinnamon cassia extract (standardized by 20% polyphenol) and fenugreek seed extract (standardized by 20% 4-hydroxyisoleucine) at 6:5:3 ratio (by weight). To prepare the cinnamon cassia extract, the cinnamon bark powder was extracted by SFT-CO_2_ to remove cinnamon bark oil. The cinnamon extract yield was measured through UV analysis, and the polyphenol content was found to be 20%. By contrast, to prepare the fenugreek seed extract, the fenugreek seeds were extracted using 55% to 75% ethanol. Moreover, the 4-hydroxyisoleucine was concentrated by strong acidic cation exchange resin. The extract was standardized by HPLC analysis of the 4-hydroxyisoleucine content, which was 20%.

### 2.3 Animals and experimental design

#### 2.3.1 Animals

Experimental Animals: male SD rats (five weeks old, 140 g to 160 g) and ICR mice (four weeks old, 18 g to 20 g) of SPF grade [Animal License No. SCXK (Beijing) 2011–0004] were supplied by SibeiFu (Beijing) Technology Co., Ltd. (China). The animals were housed at 22±1°C with 12 h light/12 h dark cycle and 50% to 60% relative humidity. Free access to food and water was provided throughout the experiment except during the fasting period, which was required by the experiments. All procedures that involve animal care were approved by the Ethics Committee of Beijing Institute of Drug Control. All animals were allowed to adapt to environment one week before the experiments. All blood samples of the rats were obtained from the vena orbitalis posterior. The animal model used in the present study was established by the protocol recommended by the China State Food and Drug Administration for the evaluation on Registered Health Foods under claims of assistant anti-hyperglycemic function [14].

#### 2.3.2 Maximum tolerance dose test

ICR mice were fasted (with free water access) overnight and randomly divided into two groups based on their body weight. These groups include a normal group and a test group, with 20 animals of half male and half female in each group. There are five animals with the same gender and group in each cage. Mice in the test group were administered with MLE at a 5 g/kg BW dose by oral gavage one time, whereas mice in the control group were given the same volume of purified water. The animals were monitored prior to dosing until 4 h after the dosing. Afterward, the animals were observed continuously for 14 days for their general status, toxicity-induced reactions, and death status. The body weights and food intake were recorded on days 1, 4, 7, 11, and 14. The animals were executed at the end of the experiments. In addition, tissues and organs were examined for changes.

#### 2.3.3 Effect of MLE on normal rats

Based on blood glucose level, 16 animals were selected from 20 healthy male SD rats and randomly assigned into normal group or treatment group, with 8 animals in each group. MLE was administered to the treatment group at 150 mg/kg BW dose, and the same volume (1.0 ml/100 g BW) of purified water was given continuously to the normal group by oral gavage everyday for 29 days. On the 30th day, the blood glucose levels were tested after the animals were fasted for 3 h with free access to water.

#### 2.3.4 Effects of MLE and MLEF on rats with hyperglycemia

The high-energy diet includes normal diet (52.6%) plus lard (10%), sucrose (15%), egg yolk powder (15%), casein (5%), sodium cholate (0.2%), calcium bicarbonate (0.6%), CaCO_3_ (0.4%), and cholesterol (1.2%). Healthy male SD rats were fed with normal diet for 4 days. They were fasted for 3 h before the blood samples were taken. The first 0 h blood samples were taken immediately after 2.5 g/kg BW dose glucose was given. The second blood samples were obtained 0.5 h after the dosing.

The blood samples were tested for changes of blood glucose levels between 0 and 0.5 h, based on which the rats were divided into five groups. These groups include the (1) normal group, (2) model group, (3) high-dose MLE treatment group (75 mg/kg BW), (4) low-dose MLE treatment group (15 mg/kg BW), and (5) MLEF treatment group (35 mg/kg BW that contains 15 mg/kg BW MLE), with 12 rats in each group.

Normal feeds were given to animals in all groups in the first week of the experiment. On the second week, high-energy diets were given to the model and treatment groups. By contrast, the normal group was given a normal diet for three weeks. Except the normal group, all the groups were administered with a single alloxan injection (ip) at 105 mg/kg BW dose after fasting with free water intake for 24 h. The animals were continuously fed with their original high-energy or normal diet for another 4 days. During the experimental period, the treatment samples were given to the treatment groups by oral gavage every day. By contrast, the normal and model groups received only the same volume of vehicle solution.

At the end of the study, the animals from all groups were fasted for 3 h. The blood samples were obtained to test the blood glucose levels (0 h). Afterward, the animals were administered with the test samples. After 15 min, glucose solution at 2.5 g/kg BW was given to the animals. Subsequently, the blood samples were obtained at 0.5 and 2 h. The levels of blood glucose (BG) at 0, 0.5, and 2 h, fasting serum insulin (FSI), cholesterol, and triglyceride were tested. In addition, the area under curve (AUC) of the glucose tolerance and insulin resistance index (IRI) was calculated using the following formula.

$${\text{AUC}}_{\text{glucose}}=\left(\text{0hBG}+\text{0.5hBG}\right)\times 0.5/2+\left(\text{2hBG}+\text{0.5hBG}\right)\times 1.5/2$$

$$\text{IRI}=\text{FSI/22}{\text{.5e}}^{\text{-ln0hBG}}\approx \frac{\text{0hBG}\times \text{FSI}}{22.5}$$

where AUC = area under curve; BG = blood glucose level (mmol/L); IRI = insulin resistance index; FSI: fasting serum insulin level.

#### 2.3.5 Preparation of the liver tissue

About 250 mg tissue sample from the left hepatic lobe of each anesthetic rat was obtained. This sample was frozen in liquid nitrogen immediately for 24 h and subsequently kept at −80°C until use.

#### 2.3.6 Statistical analysis

The data were expressed with mean ± standard deviation (M± SD), and the data were analyzed using the SPSS software (IBM, version 20.0). Briefly, the data were first subjected to normality test. If the distribution conformed to normal distribution, ANOVA was used; otherwise, nonparametric or Rank sun test was used.

### 2.4 Antibody microarray

#### 2.4.1 Antibody microarray assay

The Kinex Antibody Microarray KAM 800 (Kinexus Bioinformatics, Vancouver, B.C. Canada) analyses were performed with detergent-solubilized protein lysates as described previously [15]. Briefly, 100 μg lysate proteins from rat liver tissues were labeled with a fluorescent dye at 3 mg/mL concentration. In addition, unincorporated dye molecules were removed through ultrafiltration. Purified labeled proteins from two samples were incubated on the microarray. Each microarray consists of two identical fields. Within each field, 16 subgrids of 11 × 10 spots were used. For every 16th subgrid, positive (anti-actin, anti-tubulin, and anti-pThr antibodies) and negative control (Mouse IgG, BSA, and Print buffer) antibodies were used to optimize antibody activity, printing consistency, consistent intra- and inter-slide variabilities, and appropriate normalization of the amount of protein across different fields. Each Kinex antibody grid contains antibodies that recognize 650 signaling proteins and phosphosites. These phosphosites include about 530 pan-specific antibodies and about 270 phosphosite-specific antibodies that are intended primarily for cell signaling proteins. After probing, arrays were scanned using a ScanArray scanner (Perkin-Elmer, Wellesley, MA) with a 10 μm resolution. The resultant images were quantified using ImaGene 8.0 (BioDiscovery, ElSegundo, CA).

#### 2.4.2 Identification of genes related to MLE and MLEF treatment from antibody microarray data

The quantification of antibody microarray results was performed as described previously [16]. The quantitative analysis of the fluorescence signal strength for each target protein is performed in duplicate. First, the Z-scores for each protein (p) are calculated using the following formula:

$${\text{Z - score }=\text{ (intensity}}_{\text{P}}\;-{\text{ mean intensity}}_{\text{P1}\cdots \text{Pn}}\text{)}÷{\text{SD}}_{\text{P1}\cdots \text{Pn}}$$

where P: any one protein on the microarray; P1…Pn: all proteins on the chip; SD: standard deviations.

In the following analyses, the Z-scores for the results obtained from non-normal groups were normalized to those from the normal group. As a practice of data flooring, all Z-scores between 0 and 1 were set as -0.2, and all Z-scores between 0 and -1 were set as -0.20 to indicate that these values correspond to the absence of significant changes. The expression profiles for 19 IR-related proteins and 15 IR-non-related proteins were then subjected to clustering analysis using the ward algorithm in the absence of data standardization using JMP software tool (Research Triangle Park, NC).

#### 2.4.3 Experimental validation of the antibody microarray results through Western blot analysis

About 0.25 g of liver tissue from each rat was homogenized in a lysis buffer, which was composed of 50 mM Tris-HCl (pH 7.4), 0.1% sodium dodecyl sulfate (SDS), 2 M phenylmethylsulfonyl fluoride, and 10 g/mL leupeptin. The tissue lysate was centrifuged to remove cellular debris. In addition, protein concentration of supernatant was determined using the Pierce BCA Protein Assay Kit. Afterward, the supernatant was mixed with the reducing sample buffer for SDS-polyacrylamide gel electrophoresis (SDS-PAGE). About 20 μg protein was separated on a 12% acrylamide gel at 120 V for 60 min and blotted onto a nitrocellulose membrane. The blot was probed with a specific primary antibody and subsequently with goat anti-rabbit or mouse immunoglobulin G (IgG) conjugated with horseradish peroxidase. The blot was developed using the chemiluminescent horseradish peroxidase substrate solution (Millipore Co., Billerica, MA) for 5 min and subsequently exposed to X-ray films. The primary antibodies used were rabbit anti-JNK (catalog no. 9252, CST), anti-JRS1 (phosphoS312, catalog no. Ab66154, Abcam), and rabbit anti-PDK1 (catalog no. 3062, CST).

## Results

### 3.1 Preparation of plant materials

Using the procedure described above, the MLE powder yield was 1.85% of the weight of dry leaves. The DNJ content of MLE was found to be 5.0% through HPLC analysis. As described above, the MLEF is made up of MLE, cinnamon cassia extract, and fenugreek seed extract in a 6:5:3 ratio. The Fenugreek seed extract yield was 1.33% of the weight of dry fenugreek seeds, in which the 4-hydroxyisoleucine content was found to be 20% through HPLC analysis. The cinnamon extract yield was 5% of the weight of dry cinnamon cassia bark, which contains 20% polyphenol determined through UV analysis.

### 3.2 Acute toxicity of MLE on mice

The mice in the test group and normal group showed no abnormality within 4 h after the administration of MLE or purified water. During the 14-day observation period, no obvious abnormality was observed in animal appearance, activities, response to stimuli, secretions, and excretions. During the entire experimental period, no testing substance-induced toxic reaction or death was observed. As expected, the animal body weight in the normal group increased as well as the animals in the testing group. No statistically significant difference was observed between the body weight and food intake of the animals from the treatment and normal groups (*p* > 0.05), as shown in Tables 1 and 2. At the end of the study, all animals were dissected, and no pathological changes were observed.

**Table 1:** Body weight of mice from MLE acute toxicity study ($\bar{\text{X}}$±SD, *n* = 10).

			Body weight after treatment (g)					
Groups	Sex	Dose(g/kg BW)	Day 0	Day 1	Day 4	Day 7	Day 11	Day 14
Control	♂/♀	-/-	18.9±0.8/18.1±0.5	22.4±1.1/20.9±0.8	27.6±0.7/23.3±1.4	30.9±0.7/23.9±1.8	31.2±0.7/24.3±1.8	35.9±2.4/25.6±2.4
MLE	♂/♀	5/5	18.9±0.9/18.1±0.6	21.9±1.5/20.4±0.8	26.7±1.6/22.4±1.0	30.0±2.4/23.2±1.1	30.2±2.4/23.4±1.1	35.2±1.8/24.7±1.3

**Table 2:** Food intake of mice from MLE acute toxicity study ($\bar{X}$, n = 2).

			Body weight after treatment (g)					
Groups	Sex	Dose(g/kg BW)	Mice No.	Days 0–1	Days 1–4	Days 4–7	Days 7–11	Days 11–14
Control	♂/♀	-/-	10/10	2.8/2.9	2.3/2.7	2.1/2.0	1.6/1.5	1.8/2.1
MLE	♂/♀	5/5	10/10	2.7/2.8	2.4/2.6	2.3/2.2	1.5/1.6	1.8/2.1

### 3.3 Effect of MLE on normal rats

Oral gavage of MLE to normal rats at the dose of 150 mg/kg BW for 29 days caused no statistical difference in the fasting glucose level compared with those from the normal group. Therefore, the MLE had no effect on fasting glucose levels for normal rats (Table 3).

**Table 3:** Effect of MLE on the fasting blood glucose levels in normal rats ($\bar{\text{X}}$±SD, *n* = 8).

		Fasting blood glucose	
Groups	Dose(mg/kg)	before treatment (mmol/L)	after treatment (mmol/L)
Normal	-	6.51±0.82	7.99±0.94
MLE	150	6.59±0.75	8.43±0.61

“-“: mock control; Normal: animals fed with regular diet, MLE: animals fed with high-energy diet and treated with MLE.

### 3.4 Effects of MLE and MLEF on rats with hyperglycemia

As shown in Table 4, the BG level of rats in the control group at 0.5 h was greater than 10 mmol/L. By contrast, the fasting BG level, 0.5 and 2 h BG levels, 2 h AUC, and cholesterol and triglyceride levels in the rats from the model group were all significantly increased compared with those from the normal group (*p* < 0.001 or *p* < 0.01). These results suggested that the animal model with glucose/lipid metabolism disorder was successfully established. Meanwhile, the IRI increased in the control group compared with that in the normal group, and the difference was not statistically significant (Table 5).

**Table 4:** Effects of MLE and MLEF on blood sugar levels of rats with glucose and lipid metabolism disorders ($\bar{\text{X}}$±SD, *n* = 12).

		Blood Glucose (mmol/L)			
Groups	Doses(mg/kg BW)	0 h	0.5 h	2 h	AUC (mmol/L·h)
Normal	-	6.75±0.43**	6.60±1.28***	6.63±0.55**	13.26±1.34***
Model	-	10.11±5.42	16.12±6.07	11.00±5.58	26.90±11.21
MLE	75	7.08±2.19*	12.95±5.18	9.82±4.76	22.09±7.69
MLE	15	14.46±8.52	19.09±9.69	16.50±10.40	35.08±19.25
MLEF	35	6.48±0.56***	13.09±3.55	9.34±3.68	21.71±5.80

Note: all comparisons were conducted against those observations at similar conditions in the model group,

*** *p* < 0.001,

** *p* < 0.01,

* *p* < 0.05.

Normal: animals fed with regular diet; Model: animals fed with high-energy diet; MLE: model animals treated with MLE; MLEF: model animals treated with MLEF.

**Table 5:** Effects of MLE and MLEF on cholesterol and triglyceride levels, as well as IRI of rats with glucose and lipid metabolism disorders ($\bar{\text{X}}$±SD, *n* = 12).

Groups	Doses(mg/ke BW)	Cholesterol(mmol/L)	Triglyceride(mmol/L)	IRI
Normal	-	1.58±0.16***	0.56±0.32**	4.56±0.90
Model	-	3.32±0.86	1.12±0.48	6.10±3.12
MLE	75	2.85±0.66	1.48±0.62*	4.32±2.28
MLE	15	4.56±4.22	2.54±2.33	8.20±5.33
MLEF	35	3.32±0.35	1.22±0.75	3.81±0.89

Note: all comparisons were conducted against those at similar conditions in the control group, *** *p* < 0.001, ** *p* < 0.01, * *p* < 0.05

Normal: animals fed with regular diet; Model: animals fed with high-energy diet; MLE: model animals treated with MLE; MLEF: model animals treated with MLEF.

Compared with those of the model group, the fasting BG levels in rats from the MLE (75 g/kg BW) and MLEF (35 g/kg BW) treatment groups significantly decreased (p < 0.05 or p < 0.001). In addition, a decrease in 2 h postprandial BG levels, AUC, and serum total cholesterol levels were observed. However, the difference is not statistically significant (Tables 4 and 5). In contrast, the low dose MLE feeding did not show any anti-hyperglycemic and anti-hyperlipidemic effect in this study.

### 3.5 Investigation of the molecular mechanism using antibody array

As shown in Table 6, the expression profiles for the Erk1 (MAPK3) + Erk2 (MAPK1) proteins are identical, which indicates high data reproducibility. By contrast, the expression profiles for the ZAP70 genes are different after MLE and MLEF treatment. This outcome may result from some particular types of conformational change that occurred in the ZAP70 after the treatments. As the two antibodies may recognize different ZAP70 epitopes after the treatment, the two epitopes may have changed significantly. The affinity for the two antibodies for the ZAP70 after conformation may change significantly. However, additional experiments are needed to test this hypothesis.

**Table 6:** Expression or phosphorylation level of proteins found to be significantly changed in at least one condition. The numbers represent Z-score. Details for the explanation of the Z-score can be found in the method section. 1 and -1 are considered significantly different. The expression level of N is “0” after being normalized to itself.

Protein Name	UniProt Accession number	N	M	MLEF	MLE	IR-related
IRS1	P35568	0.00	-1.31	0.38	0.70	Yes
NFkappaB p50	P19838	0.00	-2.16	-0.17	0.23	Yes
JNK1/2/3	P45983	0.00	-1.11	0.26	-0.06	Yes
STAT5B	P51692	0.00	-1.96	0.18	0.24	Yes
MEK2	P36507	0.00	-0.20	-0.20	1.32	Yes
PTEN	P60484	0.00	-0.20	-0.40	1.11	Yes
MEK3	P46734	0.00	-0.20	0.00	1.11	Yes
mTOR	P42345	0.00	-0.20	-0.40	1.06	Yes
PKCg	P05129	0.00	0.20	-1.12	-0.98	Yes
PKCe	Q02156	0.00	0.20	-0.86	-1.11	Yes
PKCq	Q04759	0.00	0.20	-1.09	-1.39	Yes
PDK1	O15530	0.00	0.20	-1.98	-1.65	Yes
STAT3	P40763	0.00	0.20	0.00	-1.85	Yes
RafB	P15056	0.00	1.10	0.90	0.90	Yes
Hpk1	Q92918	0.00	0.20	-0.80	0.00	Yes
Erk1 (MAPK3)+ Erk2 (MAPK1)	P27361	0.00	0.20	-1.35	0.00	Yes
Erk1 (MAPK3)+ Erk2 (MAPK1)	P27361	0.00	0.20	-1.40	0.00	Yes
P53	P04637	0.00	-0.20	1.84	-0.20	Yes
Cdc42	P60953	0.00	0.20	1.68	0.40	Yes
TBK1	Q9UHD2	0.00	-0.20	1.41	1.58	No
hHR23B	P54727	0.00	-1.08	-0.88	0.72	No
S6Kb1	P23443	0.00	-0.20	2.27	1.30	No
YSK1	O00506	0.00	-0.20	1.09	1.13	No
ZAP70	P43403	0.00	-1.39	-0.01	-0.10	No
HSF4	Q9ULV5	0.00	-0.20	0.92	0.90	No
ZAP70	P43403	0.00	-1.67	-1.87	-0.60	No
Cyclin E	P24864	0.00	1.34	-0.60	0.31	No
Chk1	O14757	0.00	0.20	-1.94	-1.04	No
PED15	Q15121	0.00	-0.20	-1.40	-1.52	No
Vimentin	P08670	0.00	0.20	-1.04	-1.20	No
EGFR	P00533	0.00	-0.20	-1.87	-1.63	No
Elk-1	P19419	0.00	-0.20	-2.22	-1.70	No
c-IAP1	Q13490	0.00	1.71	-0.09	-0.03	No
*PDGFRa*	P16234	0.00	1.25	-0.45	-1.16	No

N: normal group; M: model group; MLE: model group treated with MLE; MLEF: model group treated with MLEF.

**Fig 1:**
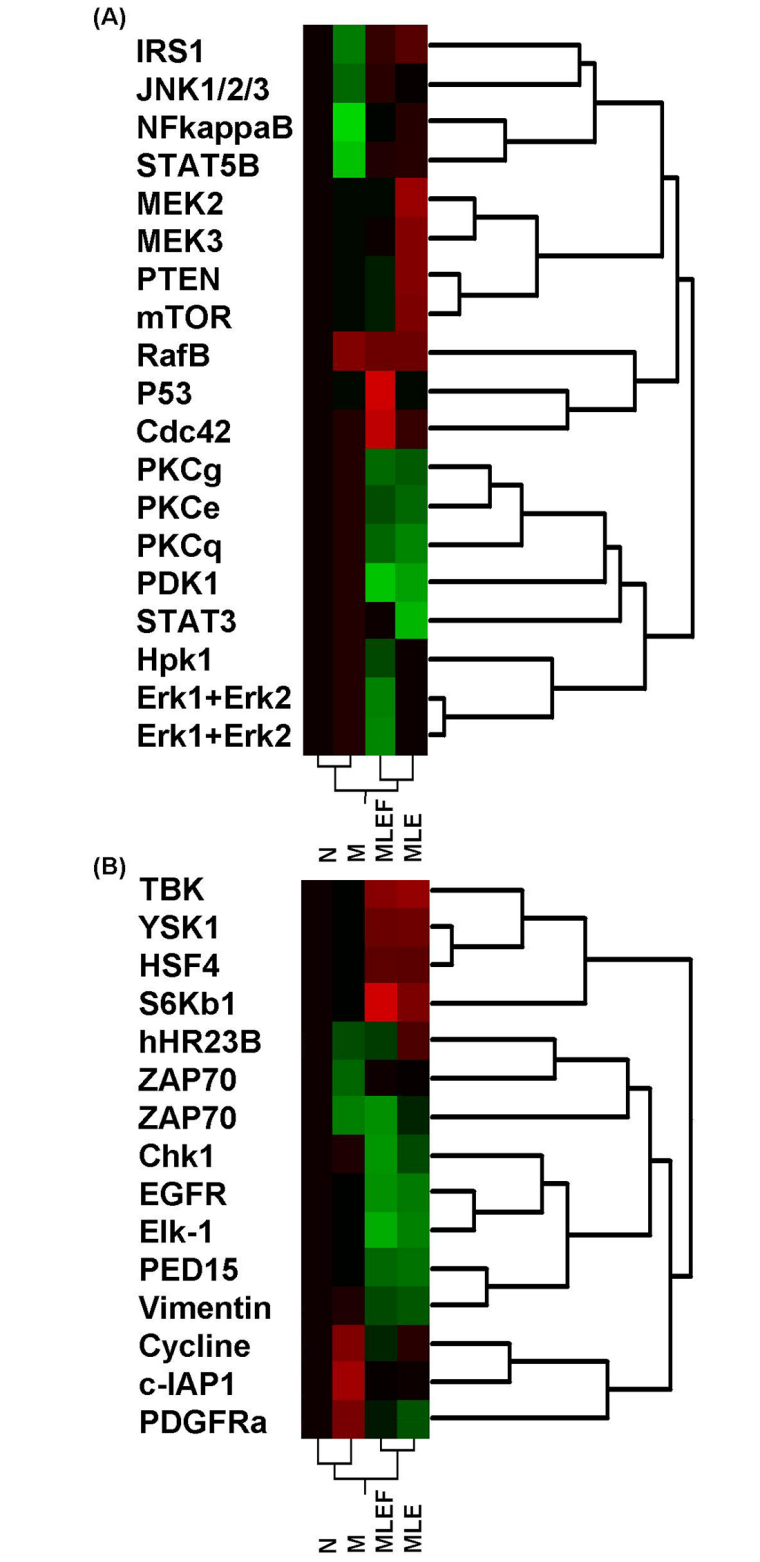
Clustering analyses of proteins that are significantly differentially expressed. N: normal; M: model; MLEF: model treated with MLEF; MLE: model treated with MLE. Black color indicates that the expression level is similar to that in the normal samples. Green color indicates that the expression level is lower compared with that in the normal sample. Red color indicates that the expression level is higher compared with that in the normal sample. (A) Proteins that are involved in the insulin-signaling pathway; (B) Proteins that are not involved in the known insulin-signaling pathway.

Notably, the antibodies used include those recognized phosphorylation sites. As a result, the increase in intensity can result from either increased expression levels or increased phosphorylation levels. For simplicity, we use “expression” to represent both “expression” and “phosphorylation.” As described above, proteins whose normalized Z-scores are > = 1 or < = −1 were considered to have differentially expressed significantly. These proteins include 19 proteins involved in IR-related pathway and 15 proteins involved in other pathways. The expression profiles for the IR-related proteins (Fig 1A) and IR-unrelated proteins (Fig 1B) across the four groups are as follows: normal, model, MLE treatment, and MLEF treatment. These groups were subjected to hierarchical clustering. These proteins show diverse expression profiles. However, two groups of proteins will be of particular interest. The first group of proteins would show expression changes in the model group and subsequent reversed expression changes after MLE and MLEF treatment. The second group of proteins would show differential expression among the MLE- and MLEF-treated groups.

As shown in Fig 1A, the expression of IRS1 (P35568), JNK1/2/3 (P45983), NFKappaB p50 (P19838), and STAT5B (P51692) are significantly downregulated in the model group. RafB was significantly upregulated, whereas Cdc42, PKCg, PKCe, PKCq, PK1, STAT3, Hpk1, and Erk1 were slightly upregulated. The expression changes of these proteins reflect the effects of the high-fat diet feeding. After MLE and MLEF treatment, the expression levels of IRS1, JNK1/2/3, NFkappaB, and STAT5B were upregulated, which was contrary to their downregulation in the model group. As a result, these proteins may be related to the MLE and MLEF effects. Similarly, the PKCg, PKCe, PKCq, and PDK1 expression levels were downregulated, which was contrary to their upregulation in the model group. The involvement of these genes in the insulin-signaling pathway is shown in S1 Fig.

Fig 1B shows that the cyclin, c-IAP1, and PDGFRa expression were upregulated, whereas those of ZAP70 and hHR23B were downregulated. The expression changes in these proteins reflect the model construction effects. After MLE and MLEF treatment, the cyclin, c-IAP1, and PDGFRa expressions were reversed compared with those seen in the model group. Therefore, this outcome may be related to the effects of MLE and MLEF treatment. Compared with proteins involved in the IR-related pathway (Fig 1A), expression changes of several proteins that were not involved in IR-related pathways are much more similar after MLE and MLEF treatment. For example, the TBK, YSK1, HSF4, and S5Kb1 expression levels were upregulated after MLE and MLEF treatment. By contrast, the EGFR, Elk-1, PED15a, and vimentin expressions were downregulated after MLE and MLEF treatment (Fig 1B).

**Fig 2:**
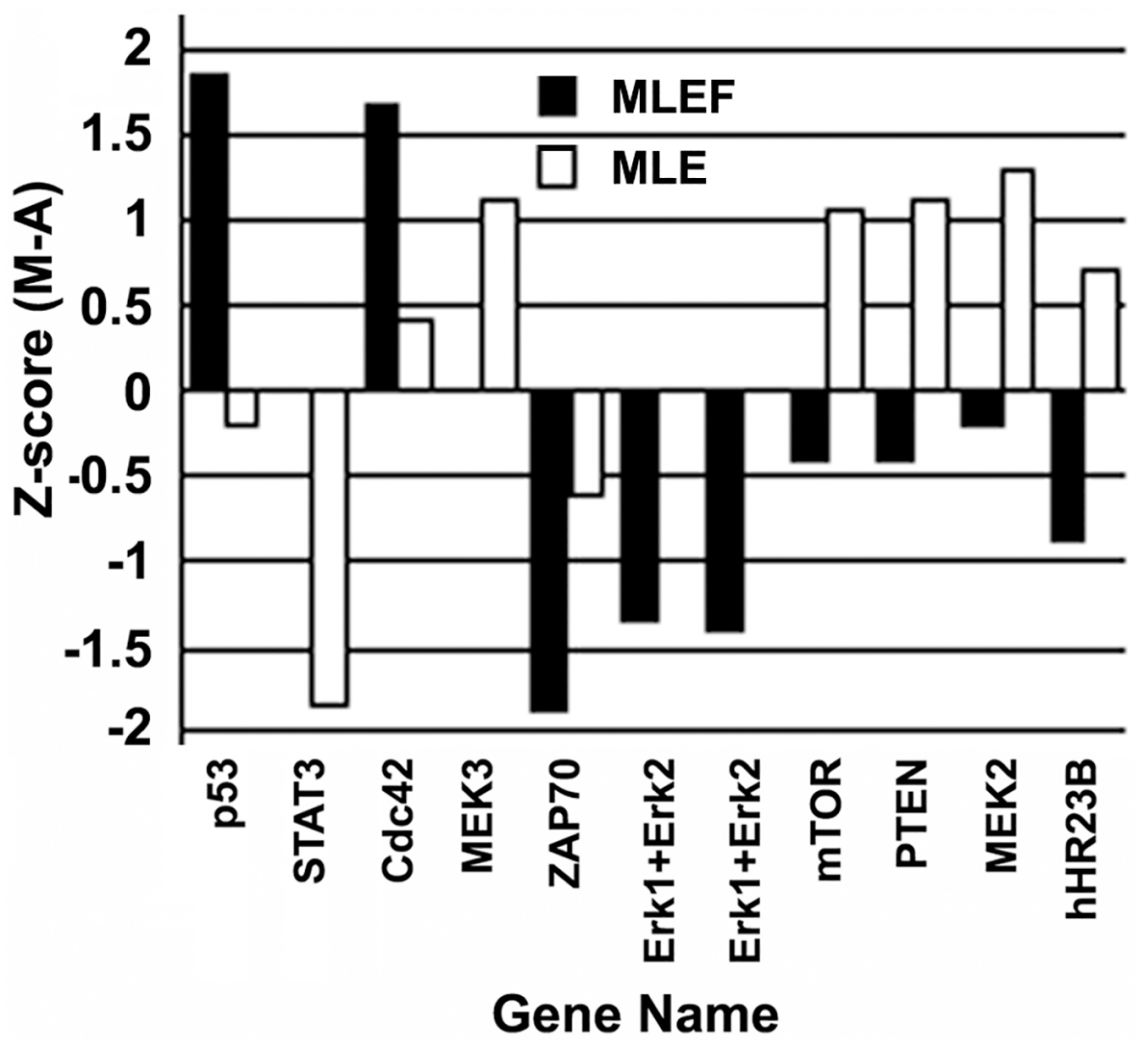
Comparison of the expression levels for proteins that are significantly differentially expressed after MLE and MLEF treatment.

To compare the effects of MLE and MLEF, the expression of proteins that were significantly differentially expressed is plotted in Fig 2. As shown in the figure, MLE treatment led to MEK3, mTOR, PTEN, MEK2, and hHR23B upregulation compared with those treated with MLEF. MLE significantly downregulated only the STAT3 expression level. By contrast, MLEF treatment led to significantly upregulated p53 and STAT3, but downregulated ZAP70, Erk, and hHR23B. The differential expressions of these proteins suggest that MLE and MLEF may have distinct effects. Interestingly, most of the affected proteins are involved in IR-related pathways (Fig 2).

### 3.6 Validation of antibody array results using Western blot analysis

Validation of protein expression levels using Western blot analysis

**Fig 3:**
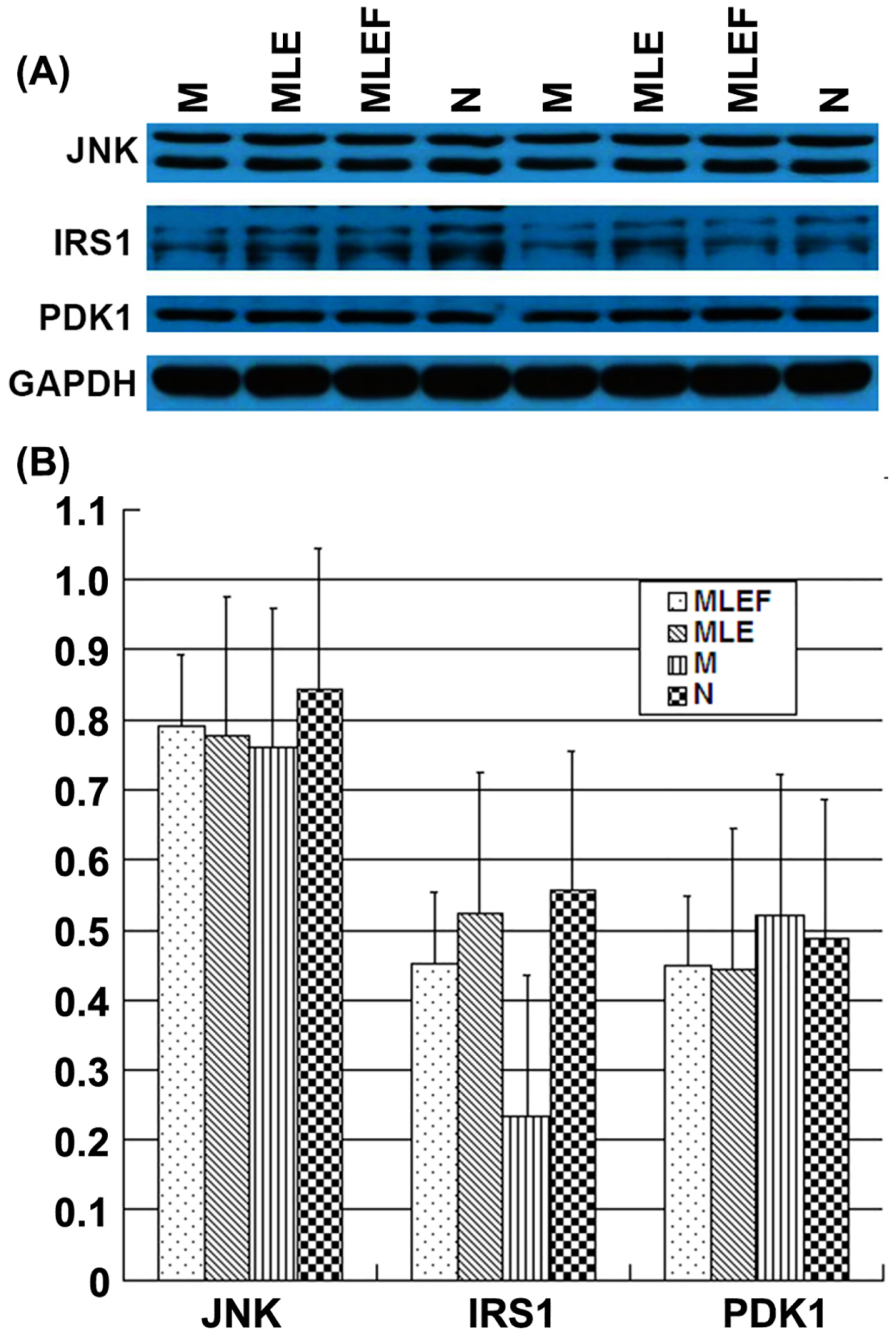
Validation of the expression levels of JNK, IRS1, and PDK1 proteins by Western blot analysis. (A) Relative expression levels of the three proteins in samples from model (M), MLE treatment (MLE), MLEF treatment (MLEF), and normal (N) animals; (B) Western blot analysis of the expression levels of JNK, IRS1 PDK1, and GAPDH in the same set of samples. GAPDH is used as internal control.

Western blot analysis of three proteins, namely, JNK, IRS1, and PDK1, was conducted, and the results are presented in Fig 3A. The experiments were performed in duplicate, with GAPDH as the control. The multiple bands seen for JNK and IRS1 are likely to represent various forms of the proteins that the antibodies recognize. The bands were then quantified using Total Lab Quant, and the results are presented in Fig 3B. As shown, among samples from the four groups, the IRS1 and JNK expression levels decreased in the model group compared with those in the normal group. In addition, their expression levels increased in the MLE and MLEF treatment groups compared with those in the model group. The expression level of PDK1increased in the model group compared with those in the normal group. By contrast, its expression decreased in the MLE and MLEF treatment groups compared with those in the model group. These results are consistent with those observed using the antibody array technology.

## Discussion

Mulberry leaf is traditionally used as food stuff in Asian countries. However, its potential toxicity and anti-hyperglycemic effects with a DNJ content of as high as 5% has never been reported in previous studies. In the current study, we have conducted a preliminary study on the toxicity and anti-hyperglycemic effects of a MLE preparation as well as a formulation of MLE with two additional herbal extracts (MLEF). Particularly, we have (1) tested the toxicity of MLE on mice; (2) tested the effects of MLE on fasting glucose levels in normal rats; (3) constructed an insulin resistance rat model; (4) tested the effects of MLE and MLEF on the rat model based on the measurement of levels of BG, cholesterol, and triglyceride and subsequently calculated the IRI; and (5) explored the potential molecular mechanism using an antibody microarray platform.

According to OECD and USEPA guidance on toxicity classification of chemicals, if the LD_50_ value is greater than 5 g/kg, then the testing substance could be regarded as non-toxic. Our results showed that the maximum tolerance dose of MLE is greater than 5 g/kg BW in mice. Therefore, MLEs are safe as food supplement.

In normal rats, the MLE treatment with up to 150 mg/kg BW for 29 days showed no effect on the fasting BG levels. By contrast, in IR model rats, the treatment with MLE (75 mg/kg BW) or MLEF (35 mg/kg), prior to and during high-energy-diet feeding and alloxan injection, significantly decreased the fasting BG levels. Trends of reduction on BG levels at the 0.5 h, 2 h, AUC of the tolerant test, cholesterol level, and IRI were also observed to be insignificant. These results indicated a potential protective effect of MLE and MLEF against high fat diet and toxic chemical-induced hyperglycemia on rats.

Interestingly, an increase in blood triglyceride levels in all treatment groups was observed compared with those in the normal rats. The increase is statistically significant for the MLE treatment at 75 mg/kg BW dose. The low dose MLE feeding did not show any anti-hyperglycemic and anti-hyperlipidemic effect in this study, on the contrary, the blood Cholesterol and triglyceride levels increased.

These results disagreed with both the general notions regarding the anti-hyperlipidemic effect of mulberry leaves and the hypolipidemic effect in the previous study [5]. One possible explanation is that the triglyceride increase might result from the long-term, high-energy feeding process. Also the large standard deviations observed in the data of low dose MLE feeding group indicated that: 1) there are large variations among the animals, 2) Low dose MLE feeding is not strong enough to eliminate the modeling effect. Additional studies need to be performed to test this hypothesis.

The animal model is critical for the correct interpretation of the obtained results. Previous studies showed that IR can be induced by long-term excessive fat and/or carbohydrate feeding in animals [16–18]. Alloxan or streptozotocine have also been used to induce diabetes in animal models; however, this kind of model is considered more relevant to insulin deficiency in type 1 diabetes or the later stage of type 2 diabetes [19].

The animal model used in the current study is established based on the method recommended by the China SFDA for the evaluation of health food products, which aims to help in the reduction of BG level. In this method, a four-week high-energy diet and a single low-dose of alloxan injection in the last week were both used to induce hyperglycemia. One advantage of this method is the shortening of the experiment period compared with those using high-energy feeding alone as stimulant. Another advantage is that this method successfully simulated symptoms of prediabetes, which is more suitable to evaluate the preventive effect of herbal remedy on diabetes mellitus. As shown in Table 4, a significant increase in the model group on the fasting BG levels, 0.5 and 2 h BG, 2 h AUC, and cholesterol and triglyceride levels were observed, which indicates the successful establishment of hyperglycemia and hyperlipidemia. However, the standard deviations of BG levels (Table 4) in the model and treatment groups were large. This finding is consistent with previous reports that alloxan injection could cause large variations on animals and absence of an adjustment process to reduce the variation; thus, this method is an evaluation for prevention effect than for treatment effect [20, 21].

One of the key questions is the potential mechanisms for the observed effects of MLE and MLEF. To answer this question, we use Kinex Antibody Microarray KAM 800 to systematically evaluate the changes in the expression or phosphorylation of a large set of cellular signaling proteins in the normal, model, and treatment groups. A schematic explanation of MLE and MLEF effects based on the results are shown in S1 Fig.

The entry of insulin signaling pathway is the insulin receptor (IR), which is a receptor tyrosine kinase that mediates the pleiotropic actions of insulin. Insulin binding leads to phosphorylation of several intracellular substrates, which includes insulin receptor substrates (IRS1, 2, 3, 4), SHC, GAB1, CBL, and other signaling intermediates [22]. Phosphorylation of IRS proteins leads to the activation of two main signaling pathways. These pathways include the PI3K-AKT/PKB pathway, which is responsible for most of the metabolic actions of insulin, and the Ras-MAPK pathway, which regulates expression of some genes and cooperates with the PI3K pathway to control cell growth and differentiation [22]. As a result, the deactivation/dephosphorylation of IRS1 would lead to the impairment of insulin effect, such as the glucose metabolism disorder and lipid metabolism disorder.

In our present study, IRS1 is the most up-stream protein in the signal pathway, whose expression levels have been found to be significantly downregulated in the model animals and upregulated after MLE and MLEF treatment (Fig 1A). As the antibody used in the array specifically recognized the phosphorylation of amino acid S312 in IRS1, the changes observed should be the IRS1 phosphorylation at S312. The regulation of IRS1 phosphorylation can lead to the following three pathways: (i) Insulin can cause the binding of the SH2 domains of PI3K to phosphotyrosines on IRS1, which leads to activation of PI3K and the generation of phosphatidylinositol-(3, 4, 5)-triphosphate (PIP3), a lipid second messenger. PIP3 activates several PIP3-dependent serine/threonine kinases, such as PDPK1, and subsequently AKT/PKB. The net effect of this pathway is to produce glucose transporter SLC2A4/GLUT4 translocation from cytoplasmic vesicles to the cell membrane to facilitate glucose transport (S1 Fig, yellow dashed line) [23]; (ii) Insulin activates Akt2 through PIP3 (red dotted line), which inhibits GSK-3 activity. GSK-3 normally inhibits glycogen synthesis, which normally stimulates glycogen synthesis. The net effect is that insulin stimulates glycogen synthesis (S1 Fig, blue dashed line) [24]; (iii) Insulin leads to inhibition of gluconeogenesis activation of Akt2 also activate SIK2, which leads to the inhibition of gluconeogenesis (S1 Fig, red dashed line) [25].

In summary, the data support the following model, (i) the treatment with high fat diet and alloxon injection inhibits IRS1 phosphorylation and activation. IRS1 activation is responsible for the stimulation of glucose transport and glycogen synthesis and inhibition of gluconeogenesis. As a result, the cells are not sensitive to insulin; (ii) treatment with MLE and MLEF upregulates the IRS1 phosphorylation to a level similar to that in the normal group. As a result, the cells recover their sensitivity to insulin.

Notably, this model does not exclude other explanation of MLE and MLEF effects through the insulin-independent signal pathway, such as the inhibition of carbohydrate metabolism and glucose absorption. Our future work on MLE and MLEF will include long-term safety study. Further evaluation is needed for the anti-diabetic effect on animal models that are more suitable than that used in the present study to observe dose vs. effect relationship. Expansion of the prevention/treatment area of MLE, such as anti-hyperlipidemia, is one of our greatest focuses.

## Supporting Information

S1 Fig A model that explains the molecular mechanism, which underlies the effects of MLE and MLEF treatment. The underlying insulin signal pathway was obtained from http://www.cellsignal.com. The yellow dashed line shows the signaling pathway that leads to GLUT4 exocytosis. The red dotted line shows Akt2 activation by PIP. The red dashed line shows the signaling pathway that leads to gluconeogenesis. The blue dashed line shows the signaling pathway that leads to glycogen synthesis.
(DOCX)

## References

[1] Lee YJ, Choi DH, Kim EJ, Kim HY, Kwon TO, Kang DG, et al Hypotensive, hypolipidemic, and vascular protective effects of Morus alba L. in rats fed an atherogenic diet. Am J Chin Med. 2011;39(1):39–52. Epub 2011/01/08. S0192415X11008634 [pii] 10.1142/S0192415X11008634 . 21213397

[2] Lim HH, Lee SO, Kim SY, Yang SJ, Lim Y. Anti-inflammatory and antiobesity effects of mulberry leaf and fruit extract on high fat diet-induced obesity. Exp Biol Med (Maywood). 2013;238(10):1160–9. Epub 2013/09/04. 10.1177/1535370213498982 1535370213498982 [pii]. . 24000381

[3] Kimura T, Nakagawa K, Kubota H, Kojima Y, Goto Y, Yamagishi K, et al Food-grade mulberry powder enriched with 1-deoxynojirimycin suppresses the elevation of postprandial blood glucose in humans. J Agric Food Chem. 2007;55(14):5869–74. Epub 2007/06/09. 10.1021/jf062680g . 17555327

[4] Naowaboot J, Pannangpetch P, Kukongviriyapan V, Prawan A, Kukongviriyapan U, Itharat A. Mulberry leaf extract stimulates glucose uptake and GLUT4 translocation in rat adipocytes. Am J Chin Med. 2012;40(1):163–75. Epub 2012/02/03. S0192415X12500139 [pii] 10.1142/S0192415X12500139 . 22298456

[5] Andallu B, Suryakantham V, Lakshmi Srikanthi B, Reddy GK. Effect of mulberry (Morus indica L.) therapy on plasma and erythrocyte membrane lipids in patients with type 2 diabetes. Clin Chim Acta. 2001;314(1–2):47–53. Epub 2001/11/24. S0009898101006325 [pii]. . 11718678 10.1016/s0009-8981(01)00632-5

[6] Jaiswal N, Maurya CK, Venkateswarlu K, Sukanya P, Srivastava AK, Narender T, et al 4-Hydroxyisoleucine stimulates glucose uptake by increasing surface GLUT4 level in skeletal muscle cells via phosphatidylinositol-3-kinase-dependent pathway. European journal of nutrition. 2012;51(7):893–8. 10.1007/s00394-012-0374-9 . 22610671

[7] Yu H, Wu M, Lu FR, Xie J, Zheng N, Qin Y, et al [Effect of Trigonella foenum-graecum 4-hydroxyisoleucine on high-glucose induced insulin resistance in 3T3-L1 adipocytes of mice]. Zhongguo Zhong xi yi jie he za zhi Zhongguo Zhongxiyi jiehe zazhi = Chinese journal of integrated traditional and Western medicine / Zhongguo Zhong xi yi jie he xue hui, Zhongguo Zhong yi yan jiu yuan zhu ban. 2013;33(10):1394–9. . 24432687

[8] Broca C, Gross R, Petit P, Sauvaire Y, Manteghetti M, Tournier M, et al 4-Hydroxyisoleucine: experimental evidence of its insulinotropic and antidiabetic properties. Am J Physiol. 1999;277(4 Pt 1):E617–23. . 10516120 10.1152/ajpendo.1999.277.4.E617

[9] Sauvaire Y, Petit P, Broca C, Manteghetti M, Baissac Y, Fernandez-Alvarez J, et al 4-Hydroxyisoleucine: a novel amino acid potentiator of insulin secretion. Diabetes. 1998;47(2):206–10. . 9519714 10.2337/diab.47.2.206

[10] Lu Z, Jia Q, Wang R, Wu X, Wu Y, Huang C, et al Hypoglycemic activities of A- and B-type procyanidin oligomer-rich extracts from different Cinnamon barks. Phytomedicine: international journal of phytotherapy and phytopharmacology. 2011;18(4):298–302. 10.1016/j.phymed.2010.08.008 . 20851586

[11] Qin B, Polansky MM, Anderson RA. Cinnamon extract regulates plasma levels of adipose-derived factors and expression of multiple genes related to carbohydrate metabolism and lipogenesis in adipose tissue of fructose-fed rats. Hormone and metabolic research = Hormon- und Stoffwechselforschung = Hormones et metabolisme. 2010;42(3):187–93. 10.1055/s-0029-1242746 . 19937569

[12] Qin B, Nagasaki M, Ren M, Bajotto G, Oshida Y, Sato Y. Cinnamon extract (traditional herb) potentiates in vivo insulin-regulated glucose utilization via enhancing insulin signaling in rats. Diabetes research and clinical practice. 2003;62(3):139–48. . 14625128 10.1016/s0168-8227(03)00173-6

[13] Verspohl EJ, Bauer K, Neddermann E. Antidiabetic effect of Cinnamomum cassia and Cinnamomum zeylanicum in vivo and in vitro. Phytotherapy research: PTR. 2005;19(3):203–6. 10.1002/ptr.1643 . 15934022

[14] Methodology for evaluating the assistant anti-hyperglycemic function [M]. publicized by China healthy food and cosmetic product department, China State Food and Drug Administration Bureau. 2012;No.107 file.

[15] Pelech S, Jelinkova L, Susor A, Zhang H, Shi X, Pavlok A, et al Antibody microarray analyses of signal transduction protein expression and phosphorylation during porcine oocyte maturation. J Proteome Res. 2008;7(7):2860–71. Epub 2008/05/20. 10.1021/pr800082a . 18484764

[16] Kobayashi Y, Miyazawa M, Kamei A, Abe K, Kojima T. Ameliorative effects of mulberry (Morus alba L.) leaves on hyperlipidemia in rats fed a high-fat diet: induction of fatty acid oxidation, inhibition of lipogenesis, and suppression of oxidative stress. Biosci Biotechnol Biochem. 2010;74(12):2385–95. Epub 2010/12/15. JST.JSTAGE/bbb/100392 [pii] 10.1271/bbb.100392 . 21150120

[17] Lim HH, Lee SO, Kim SY, Yang SJ, Lim Y. Anti-inflammatory and antiobesity effects of mulberry leaf and fruit extract on high fat diet-induced obesity. Exp Biol Med (Maywood) 2013 p. 1160–9. 24000381 10.1177/1535370213498982

[18] Bouderba S, Sanchez-Martin C, Villanueva GR, Detaille D, Koceir EA. Beneficial effects of silibinin against the progression of metabolic syndrome, increased oxidative stress, and liver steatosis in Psammomys obesus, a relevant animal model of human obesity and diabetes. J Diabetes. 2014;6(2):184–92. Epub 2013/08/21. 10.1111/1753-0407.12083 . 23953934

[19] Leiter EH, Schile A. Genetic and Pharmacologic Models for Type 1 Diabetes. Current protocols in mouse biology. 2013;3(1):9–19. 10.1002/9780470942390.mo120154 24592352 PMC3936677

[20] Andallu B, Varadacharyulu NC. Gluconeogenic substrates and hepatic gluconeogenic enzymes in streptozotocin-diabetic rats: effect of mulberry (Morus indica L.) leaves. J Med Food. 2007;10(1):41–8. 10.1089/jmf.2005.034 . 17472465

[21] Liu YN, Jung JH, Park H, Kim H. Olive leaf extract suppresses messenger RNA expression of proinflammatory cytokines and enhances insulin receptor substrate 1 expression in the rats with streptozotocin and high-fat diet-induced diabetes. Nutr Res. 2014;34(5):450–7. 10.1016/j.nutres.2014.04.007 . 24916559

[22] Fritsche L, Weigert C, Häring HU, Lehmann R. How insulin receptor substrate proteins regulate the metabolic capacity of the liver—implications for health and disease. Curr Med Chem. 2008;15(13):1316–29. 18537611 10.2174/092986708784534956

[23] Rowland AF, Fazakerley DJ, James D, E. Mapping insulin/GLUT4 circuitry. Traffic. 2011;12(6):672–81. 21401839 10.1111/j.1600-0854.2011.01178.x

[24] Shepherd PR, Navé BT, Siddle K. Insulin stimulation of glycogen synthesis and glycogen synthase activity is blocked by wortmannin and rapamycin in 3T3-L1 adipocytes: evidence for the involvement of phosphoinositide 3-kinase and p70 ribosomal protein-S6 kinase. Biochem J. 1995;305:25–8. 7826337 10.1042/bj3050025 PMC1136424

[25] Dong XC, Park S, Lin XY, Copps K, Yi XJ, White MF. Irs1 and Irs2 signaling is essential for hepatic glucose homeostasis and systemic growth. J Clin Invest. 2006;116(1):101–14. 16374520 10.1172/JCI25735 PMC1319221

