# Supplementary material for: Prevention Effects and Possible Molecular Mechanism of Mulberry Leaf Extract and its Formulation on Rats with Insulin-Insensitivity
Source: PLoS One. 2016 Apr 7;11(4):e0152728. doi: 10.1371/journal.pone.0152728 (PMC4824359; doi:10.1371/journal.pone.0152728)

Supplementary Figure 1. A model that explains the molecular mechanism, which underlies the effects of MLE and MLEF treatment. The underlying insulin signal pathway was obtained from <http://www.cellsignal.com>. The yellow dashed line shows the signaling pathway that leads to GLUT4 exocytosis. The red dotted line shows Akt2 activation by PIP. The red dashed line shows the signaling pathway that leads to gluconeogenesis. The blue dashed line shows the signaling pathway that leads to glycogen synthesis.


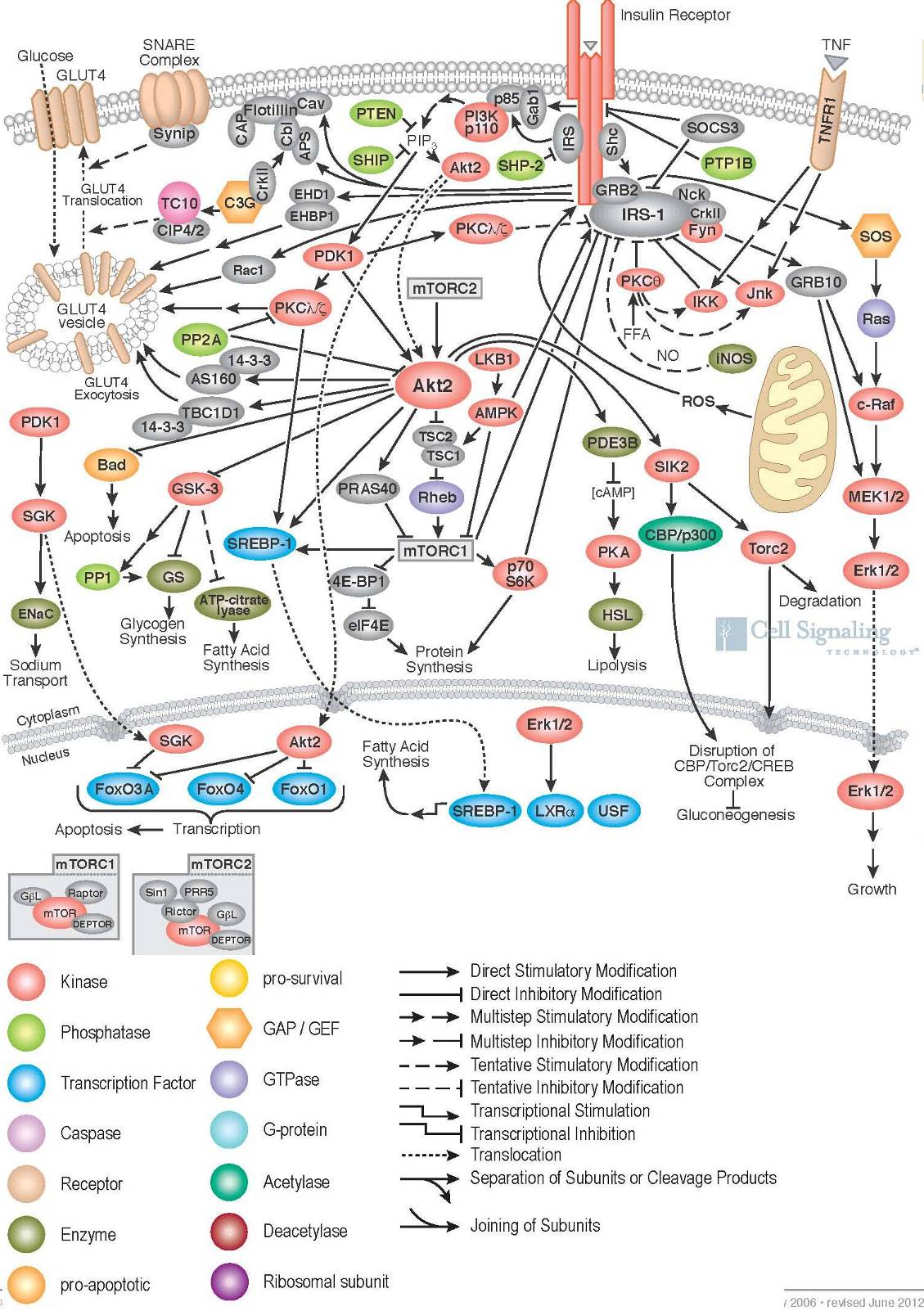

Supplement: S1 Fig — The underlying insulin signal pathway was obtained from http://www.cellsignal.com. The yellow dashed line shows the signaling pathway that leads to GLUT4 exocytosis. The red dotted line shows Akt2 activation by PIP. The red dashed line shows the signaling pathway that leads to gluconeogenesis. The blue dashed line shows the signaling pathway that leads to glycogen synthesis. (DOCX) [file pone.0152728.s001.docx]
